# Umkämpfte Globalisierung: Amerikanische und europäische Reaktionen auf Chinas Aufstieg im Hochtechnologiebereich

**DOI:** 10.1007/s11609-022-00481-x

**Published:** 2022-09-19

**Authors:** Stefan Schmalz, Helena Gräf, Philipp Köncke, Lea Schneidemesser

**Affiliations:** Staatswissenschaftliche Fakultät, Universität Erfurt (Campus), Nordhäuser Str. 63, 99089 Erfurt, Deutschland

**Keywords:** Kapitalismus, Digitalisierung, Handelskrieg, Transnationale Konzerne, Staat, Weltsystem, Capitalism, Digitalization, Trade war, Transnational companies, State, World-system, Capitalisme, Numérisation, Guerre commerciale, Firmes transnationales, État, Système-monde

## Abstract

Der Aufstieg Chinas hat zu einer Reihe von Konflikten mit den führenden westlichen Ländern geführt. Der Hintergrund dieser Auseinandersetzungen besteht darin, dass chinesische Konzerne zu ernsthaften Konkurrenten US-amerikanischer und europäischer Unternehmen geworden sind und die Ordnungsvorstellungen von US- und EU-Machteliten auf der einen und jene Chinas auf der anderen Seite auseinanderdriften. Denn in China sind politische und wirtschaftliche Macht anders organisiert als in den westlichen Marktwirtschaften: Der Parteistaat ist (Teil‑)Eigentümer wichtiger Unternehmen und interveniert offen mit Fünfjahresplänen in die Volkswirtschaft. Die Kommunistische Partei Chinas ist als Regulationsinstanz in Unternehmen präsent. Gleichzeitig organisiert der Parteistaat seine Herrschaft durch Output-Legitimität und funktioniert vielerorts als kennziffergesteuertes, meritokratisches System. Die Expansion des hybriden chinesischen Parteistaatskapitalismus führt deshalb zu einem neuen Systemkonflikt: Wirtschaftlich werden aufstrebende chinesische (Staats‑)Unternehmen zu Konkurrenten, politisch wird die Kontrolle von sensiblen Datenströmen und Infrastrukturnetzwerken zum Streitpunkt. In dem Artikel werden aktuelle Konfliktdynamiken zwischen den USA und China einerseits und der EU und China andererseits in den Bereichen Außenhandel, Investitionen, Hochtechnologie und Industriepolitik aus der Perspektive der Vergleichenden Politischen Ökonomie und des Weltsystemansatzes untersucht. Es werden jeweils Unterschiede in den Reaktionen herausgearbeitet: Die USA setzten auf eine aggressive Handels- und Sanktionspolitik, während die EU mit defensiveren Maßnahmen reagiert hat. Es wird gezeigt, wie diese Konflikte Chinas staatsgetriebene Globalisierungsstrategie verändern und zur Restrukturierung der Weltwirtschaft beitragen.

## Abstract (en)

The rise of China has led to a series of conflicts with the leading Western countries. The reasons for these disputes are that Chinese corporations have become serious competitors for US and European companies and that there are diverging approaches of governing the market between Western and Chinese power elites. In China, political and economic power are organized differently than in the US and the EU. The party-state is the (partial) owner of important companies and openly intervenes in the national economy by five-year plans. Furthermore, the Communist Party of China is engaged in companies as a regulatory authority. At the same time, the party-state organizes its rule through output legitimacy and functions in many respects as an indicator-driven, meritocratic system. The expansion of hybrid Chinese party-state capitalism is therefore leading to a new system conflict. Economically, emerging Chinese (state-owned) enterprises are becoming competitors. Politically, the control of sensitive data flows and infrastructure networks is resulting in contention. The article analyzes current conflict dynamics between the US resp. the EU and China in the areas of foreign trade, investment, high technology, and industrial policy. Comparative political economy and world-system analysis guide the analysis. In each case, there are differences in the policy responses: The US has relied on aggressive trade and sanctions policies, while the EU has reacted more defensively. It is shown how these conflicts could change China’s state-driven globalization strategy and contribute to the restructuring of the world economy.

## Résumé

L’essor de la Chine a entraîné une série de conflits avec les principaux pays occidentaux. Ces conflits s’expliquent par le fait que les groupes chinois sont devenus des concurrents sérieux pour les entreprises américaines et européennes et que les élites occidentales et chinoises ont des idées divergentes de la gestion de l’ordre du marché. En Chine le pouvoir politique et économique est organisé différemment que dans les économies de marché occidentales. L’État-parti est propriétaire (partiel) d’importantes entreprises et intervient ouvertement dans l’économie nationale par des plans quinquennaux. Le Parti communiste chinois est présent dans les entreprises en tant qu’instance de régulation. En même temps, l’État-parti organise sa domination par la légitimité que lui confèrent ses succès mesurables et fonctionne à de nombreux égards comme un système méritocratique fondé sur des indicateurs. L’expansion du capitalisme hybride chinois d’État-parti conduit donc à un nouveau conflit systémique : Sur le plan économique, les entreprises (publiques) chinoises émergentes deviennent des concurrentes, alors que sur le plan politique, le contrôle des flux de données sensibles et des réseaux d’infrastructure deviennent un point de discorde majeure. L’article examine les dynamiques des conflits actuels entre d’une part les États-Unis et la Chine, et d’autre part l’UE et la Chine dans les domaines du commerce extérieur, des investissements, des hautes technologies et de la politique industrielle. L’analyse part de la perspective de l’économie politique comparative et de l’approche du système-monde. Les différences dans les réactions sont mises en évidence : Les États-Unis s’ont focalisés sur une politique commerciale et sur des sanctions agressives, tandis que l’UE a réagi par des mesures plus défensives. L’étude montre comment ces conflits pourraient modifier la stratégie de mondialisation de la Chine et contribuer à la restructuration de l’économie mondiale.

## Einleitung

Im Mai 2020 formulierte der Ständige Ausschuss des Politbüros der Kommunistischen Partei Chinas (KPCh) das wirtschaftspolitische Ziel einer dualen Zirkulation, die später im aktuellen 14. Fünfjahresplan (2021–2025) aufgenommen wurde. Generalsekretär und Staatspräsident Xi Jinping betonte im Anschluss an die Sitzung, dass die inländische Zirkulation – sprich: die Binnenwirtschaft – nunmehr gegenüber der externen Zirkulation – sprich: der Außenwirtschaft – als Basis ökonomischer Entwicklung priorisiert werden soll. Die duale Zirkulationsstrategie verschiebt damit die wirtschaftliche Ausrichtung. Denn der Außenhandel wird in dieser neuen Konzeption als Ergänzung zur Binnenwirtschaft verstanden. Die duale Zirkulationsstrategie wird sogar als Gegenentwurf zu Deng Xiaopings Öffnungspolitik seit 1978 diskutiert (Yu 2020). Diese explizite Ausrichtung auf eine „self reliance“ bei Wertschöpfung und Technologie erinnert an Traditionslinien der Importsubstitution im chinesischen Wirtschaftsdiskurs, die bis in die Zeit Mao Zedongs zurückreichen.

Im Kern verschärft die neue Strategie Maßnahmen und Konzepte, die bereits seit der globalen Finanz- und Wirtschaftskrise 2008/09 Einzug in die chinesische Wirtschaftspolitik gehalten haben. Der Ausbau der Binnenwirtschaft und das technologische Upgrading wurden spätestens mit dem „Made in China 2025“-Plan durch eine Förderung von inländischen Zulieferstrukturen und endogener Technologieentwicklung ergänzt. Mit der dualen Zirkulationsstrategie steht nun die Ansiedlung hochwertiger Wertschöpfungssysteme im Mittelpunkt der Bemühungen der chinesischen Staatsführung. Dies bedeutet jedoch keinen Rückzug vom Weltmarkt. Vielmehr forciert der Parteistaat eine staatsgeleitete Globalisierungsstrategie mit Großprojekten wie der Belt and Road Initiative (BRI), durch die jüngst auch die Internationalisierung chinesischer Technologieunternehmen („Digitale Seidenstraße“) vorangetrieben wird. Die chinesische Staatsführung verfolgt damit einen veränderten Modus der Weltmarkteinbindung, mit dem sie Abschied nimmt vom Modell einer „verlängerten Werkbank“ der 1990er- und 2000er-Jahre, bei dem der Fokus auf dem Export von arbeitsintensiven Industriegütern lag.

Anschub für diese Reorientierung geben neue Konflikte mit den USA und im geringeren Umfang auch mit der EU. Die Regierung Trump hatte China seit 2018 mit Sanktionen und Strafzöllen überzogen, um Marktöffnungen und Deregulierungen zu erzwingen und so aus Sicht der USA „marktverzerrende Praktiken“ (Lighthizer 2020, S. 91) zu bekämpfen. Die US-Sanktionen zielen dabei insbesondere auf chinesische Technologieunternehmen wie die Netzwerkausrüster und Smartphone-Hersteller Huawei und ZTE oder Internetkonzerne wie Tencent und Bytedance. Die Regierung Biden setzt diese Politik fort und baut mit Initiativen wie dem U.S. Innovation and Competition Act of 2021 auf neue industriepolitische Ansätze. Auch die EU richtet seit 2019 seine Beziehungen mit dem chinesischen „Systemrivalen“ (Europäische Kommission 2019, S. 1) neu aus. Neben einer Reform des Investitionsregimes, das ein Screening von ausländischen Direktinvestitionen vorsieht, werden derzeit neue Regeln für staatliche Akteure aus Drittstaaten im Beschaffungswesen und bei staatlichen Subventionen diskutiert, die sich gegen chinesische Unternehmen richten. Ähnlich wie die USA verfolgt die EU-Kommission neue industriepolitische Initiativen und fördert etwa mit den Important Projects of Common European Interests (IPCEIs) grenzüberschreitende Industriekonsortien in Bereichen wie der Daten- und Cloudinfrastruktur, Batteriezellenproduktion oder der Mikroelektronik.

Die politischen Reaktionen der USA und der EU spiegeln paradoxerweise den ökonomischen Erfolg des staatskapitalistischen Modells Chinas wider. Die neue Systemkonkurrenz deutet darauf hin, dass China westliche Vorstellungen von freiem Wettbewerb und offenen Märkten missachtet, und stattdessen Instrumente zur Förderung von Technologie und konkurrenzfähigen Unternehmen entwickelt hat. Denn der chinesische Parteistaat definiert Expansionsziele in seinen Fünfjahresplänen, subventioniert Privatunternehmen, vergibt über staatliche Banken günstige Kredite und reguliert und koordiniert Investitionen. Auch deshalb ist China seit der Finanz- und Wirtschaftskrise 2008/09 zu einem wichtigen Investor in der Weltwirtschaft geworden. Die politischen Gegenreaktionen in den USA und Europa auf die Akquisitionen und Investitionen und auf den Aufstieg Chinas als High-Tech-Wettbewerber haben primär damit zu tun, dass China zu keiner grundlegenden Reform seines staatlich gesteuerten Wirtschaftsmodells und zu umfangreichen Marktöffnungen bereit ist. Dieser „clash of capitalisms“ (Milanović 2020a), bei dem sich westliche Machteliten durch das chinesische Modell herausgefordert sehen, schlägt nun auf China zurück.

Wir vertreten die These, dass die politischen Reaktionen der USA und der EU auf Chinas staatsgetriebene Globalisierung dazu beitragen, dass sich der Parteistaat nun noch stärker auf die Aufwertung der Binnenwirtschaft und die Erschließung alternativer Exportmärkte konzentriert und in der Folge eine zunehmende Blockbildung in der Weltwirtschaft nicht auszuschließen ist. Unsere Analyse zielt dabei nicht primär auf Chinas Einfluss auf internationale Organisationen (Weinhardt und Ten Brink 2020) oder Chinas Rolle auf einzelnen globalen Teilmärkten (Petry 2021), sondern thematisiert vielmehr die weitreichenden Konflikte in den chinesischen Wirtschaftsbeziehungen mit den USA und der EU sowie die daraus resultierenden Dynamiken für die Internationalisierung chinesischer Unternehmen. Wir stellen zunächst mit Bezug zur Vergleichenden Politischen Ökonomie Chinas Wirtschaftsmodell dar (Abschnitt 2). Dieses wird als hybrider Parteistaatskapitalismus beschrieben, bei dem der Parteistaat einerseits in die Wirtschaft eingreift, andererseits aber selbst Effizienzkriterien unterliegt und seine Legitimation über den Policy-Output generiert. Daraufhin wird mit Hilfe des Weltsystemansatzes die staatsgeleitete Globalisierungsstrategie und Internationalisierung chinesischer Konzerne analysiert (3). Seit 2017 lassen sich vermehrt Konflikte mit den USA und der EU um Investitionsvorhaben und den Marktzugang chinesischer Konzerne beobachten. Die unterschiedlichen politischen Reaktionen der USA und der EU auf den Aufstieg der chinesischen Wirtschaftsmacht untersuchen wir exemplarisch an den Bereichen Außenhandel, Investitionen, Hochtechnologie und Industriepolitik (4 und 5). Für diese Analyse beziehen wir u. a. Interviewdaten aus unserer empirischen Forschung ein.^1^ Abschließend wird herausgearbeitet, wie diese neuen Konfliktlinien Chinas staatsgetriebene Globalisierung und Weltmarkteinbindung zukünftig beeinflussen könnten (6).

## Hybrider Parteistaatskapitalismus in China

Die erstaunliche Performance des chinesischen Wachstumsmodells hat zu einem regen Forschungsinteresse an China beigetragen. In der Vergleichenden Politischen Ökonomie fragen heute viele Wissenschaftler:innen, welche institutionellen Regulierungen und wirtschaftspolitischen Strategien die ökonomische Entwicklung Chinas prägen und wie sie sich von jenen anderer Volkswirtschaften abgrenzen (u. a. McNally 2012; Peck und Zhang 2013; Ten Brink 2019). Diese Studien beziehen sich auf unterschiedliche Theorietraditionen – u. a. auf den Varieties-of-Capitalism-Ansatz, die Regulationstheorie und das Entwicklungsstaat-Konzept – und heben sowohl die „sichtbare Hand des Staates“ in der wirtschaftlichen Steuerung als auch private Bottom-Up-Initiativen bei der Entstehung von Zukunftsindustrien hervor. Folglich werden zur Umschreibung des chinesischen Modells höchst unterschiedliche Begriffe wie „state capitalism“ (Naughton und Tsai 2015), „state permeated market-economies“ (Nölke et al. 2019), oder „capitalism from below“ (Nee und Opper 2012) verwendet.

Trotz dieser Akzentuierungen herrscht ein weitgehendes Einvernehmen darüber, dass die staatliche Regulierung und Durchdringung der Wirtschaft in China ungeachtet der Bedeutung von Privatinitiativen und Wettbewerb stärker ausgeprägt ist als in westlichen Kapitalismusmodellen (Naughton und Tsai 2015; Lardy 2019). Dabei lassen sich unterschiedliche Stränge ausmachen: Hierzu zählen Studien, die bei Chinas Entwicklung Parallelen zu den exportgeleiteten ostasiatischen Entwicklungsstaaten Japan, Korea oder Malaysia beobachten (Breslin 1996; Knight 2014), solche, die das chinesische Wirtschaftsmodell als Teil einer Variante eines staatlich durchdrungenen oder eines Staatskapitalismus sehen, die in großen Schwellenländern wie Brasilien, Indien und Südafrika existiert (Hu et al. 2019; Nölke et al. 2019), oder wiederum solche, die die Einzigartigkeit des chinesischen Kapitalismus betonen, d. h. etwa die spezifische Rolle von öffentlichem Land- und Immobilieneigentum oder von kulturellen Praktiken wie etwa dem Einfluss von Parteikampagnen in der Ökonomie (McNally 2012; Herrmann-Pillath 2016).

Im Folgenden schlagen wir den Begriff „hybrider Parteistaatskapitalismus“ vor, um einige übergreifende Funktionslogiken des chinesischen Kapitalismusmodells zu beschreiben und die Reaktionen auf den Aufstieg Chinas besser verstehen zu können. Der Begriff bezieht sich auf einige Grundmechanismen, wie Parteistaat und Wirtschaft in China interagieren. Beim hybriden Parteistaatskapitalismus sind politische und wirtschaftliche Macht eng miteinander verzahnt. Die relative Trennung von Politik und Wirtschaft ist sehr viel diffuser als in den meisten Staaten Europas oder Nordamerikas. Branko Milanović (2020b, S. 103 ff.) bezeichnet das chinesische Modell deshalb auch in Anlehnung an Max Weber (2006 [1921/22], S. 96) als „politischen Kapitalismus“. Hierfür arbeitet er neben der kommunistischen Vergangenheit als Entstehungsvoraussetzung (a) die Rolle einer effizienten Bürokratie zur Förderung von Wirtschaftswachstum, (b) den Mangel an Rechtsstaatlichkeit und (c) eine relative Autonomie des Staates von privatwirtschaftlichen Interessen als wichtige Wesensmerkmale heraus (Milanović 2020b, S. 135 ff.). Als charakteristischen Widerspruch dieses politischen Kapitalismus identifiziert er die Notwendigkeit, Gesetze teils nur selektiv anzuwenden und den damit verbundenen Hang des Systems zur endemischen Korruption.^2^

Wir entwickeln Milanovićs Argumentation weiter, indem wir sie mit der aktuellen Debatte zur Rolle des Parteistaats in der chinesischen Wirtschaft verbinden (Pearson et al. 2020; Leutert und Eaton 2021). Unser Argument ist, dass zentrale Institutionen und Funktionslogiken der „marktsozialistischen“ Wirtschaftsordnung hybrid sind, da sie weiterhin durch das planwirtschaftliche Erbe und die Widersprüche des Parteistaats geprägt sind. Wir verstehen Hybridität daher nicht als Konzept zur Kritik an Markt-Start-Dichotomien (Underhill und Zhang 2005) oder als eine spezifische Form der staatlichen Minderheitsbeteiligung an Unternehmen (Musacchio und Lazzarini 2012). Vielmehr beobachten wir in China eine Hybridität von politischer und wirtschaftlicher Macht, durch die sich das Beziehungsgeflecht zwischen Staat und Zivilgesellschaft bzw. Politik und Ökonomie signifikant von jenem anderer Länder unterscheidet. In China ist die Wirtschaft stark durchstaatlicht und die Funktionsweise des Parteistaates ökonomisiert. Diese Eigenart spiegelt sich in zwei zentralen Merkmalen wider: nämlich in einer Staatsklasse von Kaderkapitalist:innen sowie in hybriden Institutionen des Marktsozialismus.

Hierzu ist zunächst ein staatstheoretischer Exkurs hilfreich. Die materialistische Staatstheorie hat darauf hingewiesen, dass ein Konstitutionsmerkmal des modernen bürgerlichen Staates in seiner „Besonderung“, und damit einer relativen „Trennung von Politik und Ökonomie“ besteht (Hirsch 2005, S. 21). Diese relative Trennung bedeutet, dass „die kapitalistische Gesellschaft über kein steuerndes, die Gesellschaft insgesamt umfassendes und kontrollierendes Zentrum verfügt“ (ebd., S. 38 f.). Vielmehr bilden Staat und Politik ein Terrain, in dem sich eine Vielzahl unterschiedlicher Akteure mit ihren jeweils spezifischen (und mitunter konfligierenden) Interessen und politischen Strategien aufeinander bezieht. Demokratie wird durch diese „Besonderung des Staates überhaupt erst möglich“ (ebd., S. 29), da sich die Mitglieder einer Bevölkerung nur in der politischen Sphäre des Staates als freie und gleiche Staatsbürger:innen begegnen können. Die relative Trennung von Politik und Ökonomie bedeutet aber nicht, dass die einzelnen Interessengruppen über ähnliche Einflussmöglichkeiten verfügen. Vielmehr ist der Staat asymmetrisch strukturiert und wird vom Bürgertum mit seinen hohen ökonomischen Ressourcen dominiert (Jessop 2002).

Als zentrale Einflussgruppen bildeten sich in den westlichen Industriegesellschaften Verbände als unabhängige Interessengruppen heraus: Gewerkschaftsverbände agierten als kollektive Interessensvertretung der Lohnarbeiter:innen, während die Unternehmer- und Arbeitgeberverbände als „Antiwillensgruppen“ (Cunis 1959, S. 731) operierten, um Streiks und Gewerkschaften entgegenzutreten. Solche Verbände wurden später – gerade in Kontinentaleuropa – oftmals durch korporatistische Strukturen in die Staatstätigkeit eingebunden. In China sind die Verwertungsinteressen der Unternehmen jedoch mitunter den machtpolitischen Kalkülen des Staates untergeordnet (Naughton und Tsai 2015; Nölke et al. 2019, S. 40 ff.). Der Zugang zu wirtschaftlicher Macht war lange Zeit nur über die Nähe zum Staat bzw. die aktive Mitgliedschaft in der KPCh möglich. Historisch hatte sich in China kein eigenständiges Bürgertum herausgebildet, sondern es blieb seit jeher von staatlichen Vorgaben abhängig. Zudem ist eine autonome Organisierung von Arbeitnehmer:innen kaum möglich. Die VR China kennt daher keine starken Interessenverbände. Die Staatsgewerkschaften haben nur geringen betrieblichen und politischen Einfluss; die Arbeitgeberverbände und regionalen Unternehmerverbände sind der Partei untergeordnet (Zhu und Nyland 2017).

Folglich hat sich, und das ist das *erste *Merkmal des hybriden Parteistaatskapitalismus, eine Staatsklasse von „Kaderkapitalisten“ (Overbeek 2016) herausgebildet. Anders als klassische Definitionen von rentenbasierten Staatsklassen, die sich über die Verteilung von Rohstoffrenten Legitimität erkaufen (Elsenhans 1977), ist die chinesische Staatsklasse dadurch geprägt, dass der Zugang zu politischer Macht und zum Teil sogar zu politischen Ämtern nicht primär zur Verteilung von Renten genutzt wird, sondern dem unternehmerischen Erfolg und der reibungslosen Kapitalakkumulation dient. So versammelt der nationale Volkskongress in China heute rund 100 Milliardäre, die oftmals gleichzeitig als Unternehmer:innen aktiv sind. Anders ausgedrückt: Die Top-Level-Funktionäre der KPCh sind Teil einer Staatsklasse, bei der sich politische und wirtschaftliche Macht in besonderer Weise verschränken.

*Zweitens* sind viele der Institutionen der „marktsozialistischen“ Wirtschaftsordnung hybrid. Sie bleiben durch das planwirtschaftliche, parteistaatliche Erbe geprägt. Diese Hybridität äußert sich in der Institution des (Privat‑)Eigentums: Denn der Staat ist nicht nur ein zentraler Eigentümer, der die „Kommandohöhen“ der Ökonomie und wichtige Finanzierungs- und Investitionsflüsse kontrolliert. Eigentum an Produktionsmitteln ist in China verzweigt, es existieren unterschiedliche Grade der Durchmischung von staatlichem und privatem Eigentum. Diese „Unklarheit der Eigentumsverhältnisse“ ist Milanović (2020b, S. 168) zu Folge kein „vorübergehender Zustand“ oder „Fehler“, sondern „eine grundlegende Bedingung für die Existenz“ des hybriden Parteistaatskapitalismus. Die Trennlinien zwischen verschiedenen Eigentumsverhältnissen wie Staats‑, Privat- oder Mischeigentum bleiben so auch „einigermaßen unklar“. Es existiert ein breites Spektrum hybrider Eigentumsformen, die von Kollektiveigentum bis zu privat-öffentlichen Joint Ventures reichen. Eigentum bleibt teilweise unveräußerlich – z. B. kann Grund und Boden maximal für 70 Jahre gepachtet werden – oder prekär: Bei Regelverstößen droht heute etwa Milliardär:innen bzw. Großunternehmen die (vorübergehende) Enteignung oder Zerschlagung durch die Staatsmacht.^3^

Diese Hybridität der Sozialstruktur und der Institutionen ist eng damit verbunden, dass einerseits die Ökonomie von politischen Zielen durchsetzt und damit „durchstaatlicht“, andererseits der Parteistaat durch Effizienzkriterien „ökonomisiert“ oder „rationalisiert“ ist. Die Durchstaatlichung der chinesischen Ökonomie ist dabei zwar weitgehend, lässt aber auch Freiräume für privatwirtschaftliche Initiativen (McNally 2020). Sie bezieht sich nicht nur auf den Staatsbesitz von Unternehmen in Schlüsselsektoren wie Infrastruktur, Energie oder Teilen der Industrie. Vielmehr werden durch die staatlichen Fünfjahrespläne Investitionen in Infrastruktur und einzelne Branchen gelenkt. Zum Einsatz kommen auch Förderungsinstrumente wie Subventionen oder die Kreditvergabe durch staatliche Finanzinstitutionen. Zu dieser aktiven staatlichen Industriepolitik kommen „weiche“ Formen der Steuerung hinzu. Nicht nur sind persönliche Netzwerke zwischen Staat und Unternehmen oder Parteizellen auf Unternehmensebene von Bedeutung, sondern der Parteistaat erwartet auch die aktive Teilnahme von Privatunternehmen an politischen Kampagnen, welche die Legitimität der Machtausübung der Partei untermauern. Ein Beispiel ist die aktuelle Kampagne für „Allgemeinen Wohlstand“, die ein aktives Engagement von Privatunternehmen gegen soziale Disparitäten impliziert.^4^ Diese umfangreiche Staatstätigkeit ist jedoch auch von Widersprüchen geprägt, denn der chinesische Parteistaat ist kein monolithischer Akteur: Verschiedene Gebietskörperschaften, vom Zentralstaat über die Provinzen bis zu den Bezirken, treten als wirtschaftliche Akteure und Eigentümer auf (Herrmann-Pillath 2016, S. 332 ff.). Mitunter gibt es auch Konkurrenz zwischen einzelnen Gebietskörperschaften, etwa um Investitionen.

Die Ökonomisierung bzw. Rationalisierung des Parteistaats durch Effizienzkriterien ist das Gegenstück zur Durchstaatlichung der Wirtschaft. Sie betrifft insbesondere die KPCh, mit ihren 95 Mio. Mitgliedern das Machtzentrum im chinesischen Staat. Durch klare Vorgaben und Hierarchien gekennzeichnet, fungiert sie als Scharnier für den Zugang zum staatlichen Kadersystem bzw. der öffentlichen Verwaltung und ist für Neumitglieder nur durch aufwändige Schulungen und Prüfungsverfahren zugänglich. Ungeachtet der Bedeutung von persönlichen Beziehungen, Netzwerken und Korruption ist der Parteistaat dabei an Leistungskriterien gekoppelt und hat eine meritokratische Ausrichtung. Die Karrierewege von Partei- und Staatsfunktionären stehen im engen Zusammenhang mit der erfolgreichen Erfüllung von teils verbindlichen Planzielen. Diese bezogen sich lange primär auf Wirtschafts- und Arbeitsmarktindikatoren, beinhalten heute aber auch vermehrt umwelt- und sozialpolitische Ziele. Der Parteistaat operiert somit über ein umfangreiches Kennziffersystem, das bisher eng mit der Wachstumsdynamik („GDPism“) in China verflochten war. Das politische System Chinas basiert folglich vor allem auf Output-Legitimation, was im chinesischen Fachdiskurs auch als „performance legitimation“ (Zhao 2009) bezeichnet wird. Die politische Legitimation des Parteistaats ist mit wirtschaftlichen und sozialen Erfolgen verbunden. Lange Zeit war hierfür die individuelle Aufstiegserfahrung vieler Bürger:innen zentral (Schmalz et al. 2017). Die Hybridität des chinesischen Parteistaatskapitalismus bedingt also eine wechselseitige Abhängigkeit: Nicht nur werden unmittelbare Verwertungsinteressen von Unternehmen politischen Kalkülen untergeordnet, auch ist eine möglichst reibungslose Kapitalverwertung essenziell zur Legitimierung der politischen Herrschaft des Parteistaates. Diese Legitimationsgrundlage ist indes fragil, denn die chinesische Staatsbürokratie neigt dazu, ungünstige Entwicklungen zu kaschieren oder Ziele weit auszulegen.

Zusammenfassend lässt sich feststellen, dass das hybride Verhältnis zwischen Staat und Ökonomie für den chinesischen Kapitalismus prägend ist und in den einzelnen Reformperioden immer wieder neu austariert wurde. Seit der Machtübernahme von Xi Jinping (2012 als Generalsekretär und 2013 als Staatspräsident) ist wieder eine stärkere institutionelle Verankerung des Parteistaates in der chinesischen Ökonomie zu beobachten (Pearson et al. 2020; Blanchette 2021). Diese manifestiert sich in der Gründung von Parteizellen in formal privaten Unternehmen: Nachdem Xi Jinping die Integration von Partei- und Corporate-Governance-Strukturen zur Priorität erklärte, verkündete die KPCh im Jahr 2018, dass Ende 2017 in über 73 % der privaten Unternehmen Parteigruppen gegründet wurden (KPCh 2018).^5^ Damit sind auch erweiterte Steuerungs- und Überwachungsmöglichkeiten privater Unternehmen verbunden, die die Verflechtungen zwischen Parteistaat und Unternehmen intensivieren.

## Staatsgetriebene Internationalisierung

Das chinesische Wirtschaftsmodell prägte auch die Form der Internationalisierung der chinesischen Konzerne und die Konflikte, die hieraus entstanden. Das chinesische Modell ist staatsgetrieben, aber hat sich im Laufe der Zeit gewandelt. Das rasante Wachstum der chinesischen Volkswirtschaft ging mit dem raschen Aufstieg von transnationalen chinesischen Konzernen einher, die auf die Strukturen im Weltmarkt einwirkten. Solche Aufstiegsprozesse wurden ausgiebig von den Weltsystemansätzen beschrieben, die zum Verständnis der chinesischen Internationalisierung und Globalisierung^6^ instruktiv sind. Verschiedene Theoretiker:innen beobachteten aus einer historischen Perspektive, dass der globale Kapitalismus sich immer wieder räumlich reorganisiert hat und bei diesen Umbrüchen – etwa dem Übergang von der britischen zur US-Hegemonie ab dem späten 19. Jahrhundert – (geo)ökonomische Prozesse mit (geo)politischen Veränderungen im Staatensystem interagierten (Hopkins und Wallerstein 1982, S. 104 ff.; Arrighi 2003). Solche hegemonialen Übergänge brachten die Globalisierung der Weltwirtschaft jeweils unter der Ägide eines Hegemonialstaats, wie etwa in der liberalen Weltwirtschaftsordnung der Pax Britannica Ende des 19. Jahrhundert, auf ein neues Niveau, führten dann aber mit dem Niedergang einer hegemonialen Konstellation zu Phasen von Staatenkonflikten und Deglobalisierung, die schließlich jeweils mit einem „hegemonic breakdown“ endeten (Arrighi und Silver 1999, S. 33; Chase-Dunn et al. 2000).

Die Hegemonialmächte bauten ihre politische und ökonomische Macht auf umfangreichen globalen Strukturen auf. Die USA waren seit dem Zweiten Weltkrieg die unumstritten größte Volkswirtschaft, ein zentraler Finanzplatz und beherbergten die wichtigsten institutionellen Schaltstellen der Weltwirtschaft wie die Federal Reserve. Darüber hinaus kontrollierten sie weitere wichtige Machtstrukturen: So spielten nach dem Zweiten Weltkrieg neben dem mit dem Namen Bretton Woods verbundenen globalen finanzpolitischen Institutionengefüge insbesondere jene US-Konzerne eine wichtige Rolle, die ein globales Netzwerk mit umfangreichen Shareholder-Beteiligungen in der EU und anderen Weltregionen gebildet hatten und die globalen Märkte dominierten (Starrs 2013). In ähnlicher Weise stehen heute die Tech Five Apple, Microsoft, Alphabet, Facebook und Amazon an der Spitze der globalen Wertschöpfung und erwirtschaften hohe Profitraten. Die aktuelle Globalisierungsperiode wurde daher bisher von US-amerikanischen Unternehmen dominiert (Panitch und Gindin 2012).

Der ökonomische Aufstieg Chinas fordert diese US-Dominanz zunehmend heraus. Chinesische Unternehmen waren lange Zeit ausschließlich auf den Binnenmarkt orientiert und verfügten, anders als ihre US-amerikanischen, europäischen und japanischen Konkurrenten, über kein globales Netzwerk, obgleich das chinesische Wirtschaftsmodell bereits in den 1990er-Jahren hochgradig transnationalisiert war. Neben der dynamischen Binnenwirtschaft spielten der Export und der Zufluss von ausländischen Direktinvestitionen eine wichtige Rolle bei den Entwicklungserfolgen. Hohe Exportüberschüsse trugen zur Erwirtschaftung von Devisen bei, Investitionszuflüsse konnten für den Technologietransfer genutzt werden. Der chinesische Exportsektor wurde von ausländischen Konzernen dominiert. Ausgeführt wurden bis in die 2000er-Jahre vor allem zu Niedriglöhnen produzierte Industrie- und Konsumgüter, die oftmals nur bloße Re-Exporte von importierten Komponenten oder Halbfertigwaren waren („export-processing-Produktion“). Das chinesische Modell galt jedoch als unausgewogen und wurde oftmals als „verlängerte Werkbank der Welt“ beschrieben. Chinas Weltmarktintegration war eine wichtige Voraussetzung für den Neoliberalismus in den OECD-Staaten. Die Auslagerungen und preiswerten Importe begünstigten etwa die träge Reallohnentwicklung der US-Ökonomie (Liew 2010). Diese Beziehungen zwischen den USA und China wurden sogar mitunter als symbiotisch wahrgenommen und entsprechend von Niall Ferguson als „Chimerica“ bezeichnet (Ferguson 2009, S. 294 ff.) – mit China als Exportnation und Werkbank der Welt einerseits und den USA als verschuldetem und defizitärem „consumer of last resort“ andererseits.

Mit dem wirtschaftlichen Aufstieg rückte China in eine immer bedeutendere Stellung. Nach den Turbulenzen der globalen Finanz- und Wirtschaftskrise wuchs es zur zweitgrößten Volkswirtschaft der Welt heran. Allerdings forderte China in dieser ersten Phase seiner Internationalisierung die bestehenden globalen Machtstrukturen nicht offensiv heraus. Zwar begann der chinesische Staat mit der Going-Global-Strategy ausländische Investitionen ab 1999 systematisch zu fördern (Schmalz 2019, S. 23 ff.). Dies führte aber zunächst zu keiner Konfrontation mit den USA und ihren Verbündeten. Die zentrale Motivation bestand in der Sicherung von Ressourcen: Nach Zahlen des Global China Investment Trackers flossen zwischen 2005 und 2007 rund 31,7 % aller chinesischen Direktinvestitionen in den Energiesektor und 19,6 % in den Abbau von Industriemetallen (American Enterprise Institute und Heritage Foundation 2021). Auch Infrastrukturinvestitionen wurden getätigt, meist im Tausch gegen langfristige Rohöl- oder Rohstofflieferungen („Angola-Modell“). Diese Investitionen erfolgten bereits in dieser ersten Phase in Form einer „statist globalization“ (Harris 2009). Hierbei handelte es sich überwiegend um Staatskonzerne, die politisch sensible Akquisitionen in den USA oder der EU vermieden. Auch chinesische Finanzinstitutionen wie die China Development Bank waren an der Finanzierung der Vorhaben beteiligt. Der Hauptanteil floss in Greenfield-Investitionen in Länder des globalen Südens wie Angola, Brasilien oder den Iran. Auch wenn die ausgehenden Direktinvestitionen auf 30,5 Mrd. US-Dollar im Jahr 2007 wuchsen und mit der Gründung des Staatsfonds China Investment Corporation im selben Jahr einen weiteren Schub erhielten, flossen sie primär in vorgelagerte Stufen der globalen Wertschöpfungsketten. Die Mittel, die der Parteistaat zur Förderung von Auslandsinvestitionen vergab, blieben gering. Die chinesische Zentralbank investierte Exportüberschüsse und Devisen stattdessen primär in den US-(Staats‑)Anleihemarkt: Im Jahr 2007 hielt die Treasury Bonds über 477,6 Mrd. US-Dollar. Diese Kapitalzuflüsse in die Anlagemärkte waren die finanzielle Komponente des Chimerica-Modells. Die stetigen Zuflüsse stützten die Niedrigzinspolitik der FED, heizten den US-Konsum an und festigten den Status Quo. Als Folge blieben die chinesischen Investitionsbestände im Ausland gering.

Die globale Finanz- und Wirtschaftskrise 2008 leitete die zweite Phase der chinesischen Internationalisierung ein. Grundlage hierfür waren die Auswirkungen der Krise auf die chinesische Exportwirtschaft. Das bestehende Chimerica-Modell wurde erschüttert. Dem massiven wirtschaftlichen Einbruch begegnete der Parteistaat mit einem Konjunkturprogramm im Umfang von 586 Mrd. US-Dollar. In der Folge forcierten viele Maßnahmen ein Re-Balancing der chinesischen Volkswirtschaft. Diese Maßnahmen zielten darauf, mittelfristig wirtschaftliche Aktivitäten mit höherer Wertschöpfung in China anzusiedeln, die Industrieproduktion aufzuwerten, den Binnenmarkt auszubauen und das Exportregime mit seinen niedrigen Löhnen aufzubrechen (zur Diskussion vgl. Ahuja et al. 2013; Hung 2016).

Außenwirtschaftlich zog dies weitgehende Veränderungen nach sich. Die Partei- und Staatsführung begann von der einseitigen Orientierung auf die Märkte der USA, EU und Japans abzurücken und forcierte die wirtschaftliche Kooperation mit Staaten des Globalen Südens, etwa in der 2009 gegründeten BRIC(S)-Gruppe. Im Folgejahrzehnt nahm der Anteil der G7-Staaten am chinesischen Export von 39,1 % (2007) auf 34,1 % (2016) ab (Schmalz 2018, S. 322). Bei den Auslandsinvestitionen stand die Parteiführung unter starkem Rechtfertigungsdruck, da staatliche chinesische Finanzinstitutionen bis zu 400 Mrd. US-Dollar in die US-amerikanischen Immobilienbanken Fannie Mae und Freddie Mac investiert hatten, die vor der Pleite standen (ibid., S. 205). Die Fehlinvestitionen trugen zu einem Umsteuern bei. Nach der Finanzkrise setzten chinesische Investoren auf eine Diversifizierung ihrer Anlagen. Sie forcierten ausländische Direktinvestitionen, die nun auch oft in die EU und die USA flossen. Es kam vermehrt zu Übernahmen: Anfänglich nutzten die Anleger die Krise als Gelegenheitsfenster, um angeschlagene Unternehmen zu akquirieren, später wurden die Investitionen staatlich koordiniert.

Die „Made in China 2025“-Strategie leitete chinesische Anleger:innen dazu an, verstärkt in den Erwerb von Technologie zu investieren, um die industrielle Aufwertung der Volkswirtschaft zu unterstützen. Im Jahr 2015 war Europa erstmals die wichtigste Zielregion für chinesische Direktinvestitionen. Einer Studie von MERICS und der Rhodium-Gruppe zufolge (Wübbeke et al. 2016) gingen im Jahr 2016 rund 35 Mrd. Euro in die EU, die Hälfte der Gelder floss dabei in Akquisitionen in Sektoren, die in „Made in China 2025“ als strategisch identifiziert wurden. Für 2014 bis 2017 lassen sich 64 % der chinesischen Beteiligungen an deutschen Unternehmen mit einem Eigenkapitalanteil von mindestens 10 % diesen Schlüsselindustrien zuordnen (Jungbluth 2018). Dabei spielten auch private Investoren eine größere Rolle, deren Akquisitionen jedoch staatlich gefördert wurden. Die regionale Diversifizierung bedeutete indes nicht, dass andere Weltregionen an strategischer Bedeutung verloren: So wurden etwa mit der Belt-and-Road-Initiative große Summen in Infrastrukturprojekte in Asien investiert, wie auch in Afrika und Lateinamerika. China stieg so zum zweitgrößten Investor weltweit auf und tätigte zwischen 2008 und 2017 Direktinvestitionen von rund 1,7 Bio. US-Dollar (American Enterprise Institute und Heritage Foundation 2021). In nur einem Jahrzehnt erreichten die Direktinvestitionsbestände im Ausland einen Umfang vergleichbar mit jenen Deutschlands. China wurde somit zu einem aktiven Treiber einer staatsgeleiteten Globalisierung.^7^

Doch bereits Ende 2016 musste die chinesische Staats- und Parteiführung erneut umsteuern (Schmalz 2019, S. 28 ff.), womit eine dritte Phase der chinesischen Internationalisierung begann. Zuvor, im Sommer 2015, war es zu einem Börsencrash gekommen: Die disproportionale ökonomische Entwicklung auf dem Binnenmarkt hatte zu Überinvestitionstendenzen und einer hohen Verschuldung von Kommunen und Unternehmen beigetragen (Pauls 2015). Nach dem Börsencrash folgten Kapitalabflüsse, die hohe dreistellige Milliardensummen erreichten. Die Kapitalflucht war oft mit Investitionen im Finanz- und Immobiliensektor verbunden, über die – wie etwa beim Versicherungskonzern Anbang (s. Fn. 3) – große Summen ins Ausland verschoben wurden. Die Regierung verhängte daraufhin Ende 2016 neue Prüfungsverfahren und Regulierungen für größere Investitionsvorhaben im Ausland. Gleichzeitig begann eine Kampagne gegen vermögende Chines:innen, die Regeln umgangen hatten. Die Trendwende wurde durch die Probleme im Finanzsektor verschärft, da nun die Bereitschaft im chinesischen Bankensektor sank, größere Investitionsvorhaben im Ausland zu finanzieren. Als Folge sanken die chinesischen Direktinvestitionen ab 2017 deutlich.

**Figure 1:**
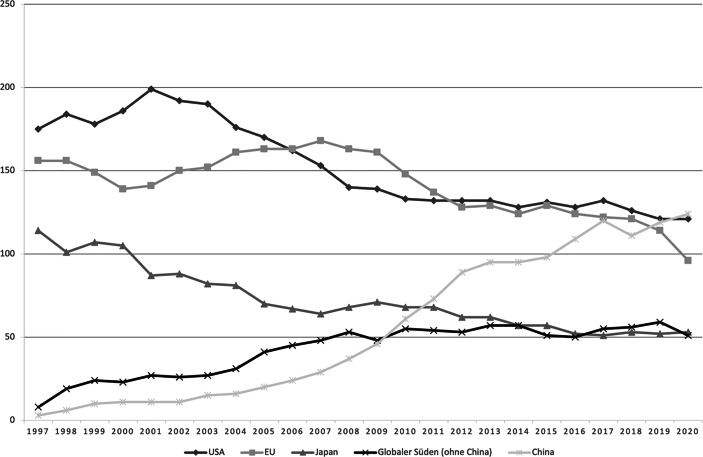

Ungeachtet dieses Rückgangs waren viele chinesische Konzerne zu Weltmarktführern aufgestiegen (vgl. Abb. 1). Im Jahr 2020 befanden sich mit 124 mehr Unternehmen aus China unter den 500 umsatzstärksten Unternehmen der Welt als aus den USA mit 121 oder der EU mit 96 – zur Jahrtausendwende waren es noch 11 und 2010 61 Unternehmen. Zudem hatte China die USA bereits 2019 als größte Exportnation überholt und war nun zum zweitgrößten Investor weltweit aufgestiegen, sodass chinesische Konzerne heute hochgradig internationalisierte Unternehmensnetzwerke unterhalten. Bei den großen chinesischen Konzernen handelt sich in vielen Fällen um wettbewerbsfähige Technologieunternehmen. Unter den Top 500 finden sich etwa Smartphonehersteller und Netzwerkausrüster wie Huawei und Xiaomi, Automobilkonzerne wie Geely und Elektronikkonzerne wie Midea. Die meisten Großkonzerne weisen dabei enge Verbindungen zum chinesischen Parteistaat auf. In vielen Fällen, wie beim Automobilgiganten SAIC, ist der Staat Eigentümer, oder aber die CEOs sind eng mit der KPCh vernetzt, wie im Fall des Marktführers für Haushaltsgeräte Haier.

Die zunehmende wirtschaftliche Konkurrenz mit chinesischen Wettbewerbern und die Angst vor staatlicher Einflussnahme trugen seit 2017 zu unterschiedlichen politischen Reaktionen in den USA und der EU bei. Denn sowohl die damals neu gewählte US-Regierung Trump (2017–2021) als auch die EU-Kommission Juncker (2014–2019) begannen, chinesische Investitionen und Konzernaktivitäten kritisch zu hinterfragen und verhängten neue außenwirtschaftliche Maßnahmen. Die dritte Phase der chinesischen Internationalisierung äußert sich darum auch in neuen Konfliktlinien in der globalisierten Ökonomie. Diese Konflikte sind insbesondere in drei Bereichen sichtbar: im Bereich der Investitionsregulierung, in der Außenhandelspolitik und in der Hochtechnologie-Konkurrenz; hinzu kommen neue industriepolitische Initiativen. Die Regulierungsinitiativen in den USA und der EU werden in den folgenden zwei Abschnitten vergleichend untersucht.

## Die USA seit Trump: High-Tech-Wirtschaftskrieg und strategisches Decoupling

Die außenwirtschaftliche Reaktion der Regierung Trump auf die aufstrebenden chinesischen Konzerne war weitgehend. Sie umfasste einen Mix aus Importzöllen, Regulierungen von Investitionen, Sanktionen gegenüber chinesischen Technologieunternehmen, industriepolitischen Maßnahmen und sogar neuen Regeln im Finanzsektor. Damit wurden die engen wirtschaftlichen Beziehungen zwischen den USA und China, und damit das bereits beschriebene „Chimerica“-Modell, grundsätzlich infrage gestellt. Nachdem die chinesische Staats- und Parteiführung hiervon bereits abgerückt war – etwa indem sie den Ankauf von US-Staatsanleihen beendet hatte –, ging die Kritik der Trump-Administration am bewährten Modell über die bloße Verlagerung von Arbeitsplätzen hinaus, indem sie vor allem auf Chinas staatsgetriebenes Wirtschaftsmodell und damit insbesondere auf den staatlich protegierten Hochtechnologiesektor zielte (Lighthizer 2020; Scherrer 2021, S. 60 ff.). Diese Neuorientierung wurde von unterschiedlichen Gruppen in der Regierung gestützt, darunter Vertreter:innen des traditionellen republikanischen Parteiestablishments (z. B. Robert Lighthizer), die sich einen härteren Umgang mit China wünschten, und neue Akteure in der Trump-Administration wie Steve Bannon oder Peter Navarro, die rechtsnationalistische und protektionistische wirtschaftspolitische Vorstellungen einbrachten. Für einen härteren Umgang mit China erhielt die Regierung aber auch Unterstützung aus der Bürokratie im State Department oder dem Office of the United States Trade Representative sowie – anders als bei den meisten anderen Politikfeldern – aus großen Teilen der Demokratischen Partei.

Der Kurs gegenüber China war folglich eine Form des „Techno-Nationalismus“ (Samuels 1994; vgl. auch Starrs und Germann 2021), bei dem China durch Angriffszölle und Sanktionen zum Einlenken gebracht werden sollte. Diese Maßnahmen richteten sich gegen die Funktionsweise des hybriden Parteistaatskapitalismus in China, der bisher mit Zöllen, Subventionen, Kapitalverkehrskontrollen und Rahmenplänen erfolgreich zur technologischen Entwicklung des Landes beigetragen hatte. Im Zentrum standen Forderungen nach einer Marktöffnung für US-Exporte, dem Schutz intellektuellen Eigentums sowie einem staatlichen Förderungsstopp von Zukunftsbranchen wie der Chipindustrie (Scherrer 2021, S. 60 ff.; Schmalz 2021). Die Zielsetzung bestand folglich nicht nur in einer Korrektur der Handelsbilanz oder einer Dynamisierung der heimischen Industrie, sondern vor allem darin, in Zukunftsbranchen die Führungsrolle zu behalten. Das Drehbuch für die Konfrontation folgte der Politik der Regierung Reagan gegenüber Japan, das in den 1980er Jahren ebenfalls deutliche Handelsbilanzüberschüsse mit den USA erwirtschaftet und Fortschritte in der Hochtechnologie gemacht hatte. Damals wurden mit Hilfe des Abschnitts 301 des Handelsgesetzes aus dem Jahr 1974 Importrestriktionen und hohe Zölle gegen Güter wie Autos verhängt und erfolgreich Wettbewerbsvorteile für die US-Industrie abgesichert (Gilpin 2000, S. 235 f.).

Als Hegemonialmacht setzte die Regierung Trump in der Konfrontation mit China erneut auf ihre „strukturelle Macht“ und damit die Fähigkeit, Spielregeln in der Weltwirtschaft zu gestalten und diese auch in den bilateralen Wirtschaftsbeziehungen mit China auszunutzen. Ihre Maßnahmen bezogen sich auf folgende Bereiche:

### Außenhandel

Ab Juli 2018 verhängte die Regierung Trump stufenweise umfangreiche Importzölle, die Anfang 2020 rund 70 % der US-Importe aus China betrafen und von chinesischer Seite jeweils mit Gegenmaßnahmen beantwortet wurden. Der Durchschnittszoll für Importe aus China stieg so von 3,1 % (2018) auf 19,3 % (2022) an, die Importzölle für amerikanische Produkte wuchsen im selben Zeitraum von 8 % auf 21,2 % (Bown 2022). Diese Zölle wirkten sich nicht nur auf chinesische Exporteure und Investoren aus, die auf den US-Markt ausgerichtet waren. Vielmehr entschieden verschiedene ausländische Konzerne, die China als verlängerte Werkbank nutzten, Teile ihrer Produktion innerhalb Ostasiens auszulagern („China plus One“-Strategie). Chinas Rolle als Exporteur und als Zulieferer für Firmen in US-zentrierten Produktionsnetzwerken wurde nun für das Land zum Problem. Gleichzeitig ergaben sich Friktionen im bilateralen Warenhandel.

### Investitionen

Mit dem Foreign Investment Risk Review Modernization Act verschärfte die US-Regierung im Jahr 2018 zudem die Überprüfung ausländischer Investitionen, die dem Committee on Foreign Investment in the US (CFIUS) obliegt. Neben einer Budgetaufstockung wurden die bereits weitgehenden Kompetenzen deutlich ausgeweitet:

Whereas before CFIUS could block investment leading to control of a firm in certain proscribed sectors relating to national security, it could now scrutinize any investment in technologies deemed to be “foundational” and “emerging”, any investment at all from SOEs, as well as real estate transactions close to military installations. (Starrs und Germann 2021, S. 1128)

Zusammen mit den stärkeren Kontrollen in China führte die Reform zu einem massiven Rückgang chinesischer Direktinvestitionen um über 80 %. Bereits seit 2016 wurden gerade in der Halbleiterbranche immer wieder Übernahmen von und Beteiligungen bei Firmen wie Lumileds, Fairchild Semiconductor und Magnachip blockiert. CFIUS verhinderte nach der Reform zudem verschiedene Beteiligungen an Plattform-Unternehmen wie die Gesundheitscommunity-App PatientsLikeMe, die Hotelmanagement-Plattform StayNTouch oder die Gay-Dating-App Grindr, die nur schwer mit nationalen Sicherheitsinteressen in Verbindung gebracht werden können.

### Hochtechnologie-Konkurrenz

Außerdem wurden chinesische Tech-Firmen mit der Hilfe von Exportverboten (Export Control Reform Act) ins Visier genommen. Die US-Regierung lastete zunächst dem Telekommunikationsunternehmen ZTE und später Huawei einen Verstoß gegen die Iran-Sanktionen an. Beide Unternehmen hatten sich zu bedeutenden Wettbewerbern im High-Tech-Sektor aufgeschwungen. Huawei dominierte etwa den Weltmarkt für 5G-Netzwerke und Smartphones. Dabei ging die US-Regierung so weit, dass ZTE zeitweise seine Produktion einstellen musste, da der Konzern keine Halbleiter aus den USA mehr beziehen konnte. Letztlich musste das Unternehmen seine Geschäftsführung austauschen und sich unter Aufsicht eines US-amerikanischen Compliance-Teams stellen (ebd., S. 1129).^8^ Für diese Politik nutzten die USA die hohe Abhängigkeit der chinesischen Tech-Konzerne von US-amerikanischen Halbleiter-Importen aus. Huawei wurde sogar auf eine schwarze Liste mit Unternehmen gesetzt, die ein Sicherheitsrisiko darstellen, sodass der Konzern z. B. keine Google-Anwendungen mehr auf neuen Handymodellen mitausliefern kann und mit der Huawei AppGallery einen eigenen App-Store aufbauen musste. Mit dem Argument, die nationale Sicherheit schützen zu müssen, sanktionierte die Regierung Trump eine ganze Reihe chinesischer Unternehmen. Hierzu gehören auch der chinesische Chiphersteller SMIC, der beschuldigt wird, zivile Technologien für militärische Zwecke zu nutzen sowie der chinesische Drohnenhersteller DJI, dem Menschenrechtverletzungen vorgeworfen werden. Auch die Regierung Biden griff immer wieder auf den Export Control Reform Act zurück, etwa wegen Menschenrechtsverletzungen in der Provinz Xinjiang. Heute existiert daher eine umfangreiche schwarze Liste mit Export- und Investitionsbeschränkungen für chinesische Konzerne. Allein die Liste mit Exportbeschränkungen umfasste im April 2022 183 Seiten mit Dutzenden chinesischen Organisationen und High-Tech-Konzernen.^9^ Hinzu kamen weitere Maßnahmen wie die restriktivere Visa-Vergabe für chinesische Studierende oder der Holding Foreign Companies Accountable Act, durch den die Börsennotierung von ausländischen Unternehmen in den USA an striktere Transparenz- und Kontrollstandards gebunden wird (Bloomberg 2019; Swanson und Bradsher 2020).

Der Druck auf die chinesische Regierung erhöhte sich somit stetig, aber die Maßnahmen reichten nicht aus, um sie zu einer weitgehenden Marktöffnung oder gar Reform des chinesischen Wirtschaftsmodells zu zwingen. Vielmehr geriet die Regierung Trump vor dem Hintergrund des Wahlkampfs 2020 durch die chinesischen Gegenzölle – insbesondere auf landwirtschaftliche Produkte – und die steigenden Importkosten selbst unter Druck, sodass die USA und China im Januar 2020 ein Zwischenabkommen („Phase One Deal“) mit graduellen Zollerleichterungen für chinesische Importgüter im Wert von 120 Mrd. US-Dollar und einer Zusage zur zusätzlichen Abnahme von US-Gütern über 200 Mrd. US-Dollar durch China abschlossen.

Die seit 2021 amtierende Regierung Biden behielt die Grundorientierung eines konfrontativen außenwirtschaftlichen Umgangs mit China bei. Die außenhandelspolitischen Weichenstellungen der Regierung Trump gegen China wurden bisher nicht korrigiert (Scherrer 2021, S. 309 ff.). Allerdings setzte die Regierung Biden auf eine aktive Industriepolitik im Hochtechnologiesektor: Mit dem U.S. Innovation and Competition Act of 2021 werden rund 250 Mrd. US-Dollar in Schlüsseltechnologien wie KI-Forschung oder Biotechnologie investiert, um die US-Wirtschaft gegen die aufstrebende chinesische Konkurrenz zu wappnen. Dabei baut die Regierung Biden auf einige Traditionslinien der US-amerikanischen Industriepolitik, die auf Netzwerkbildung zwischen Staat, Wissenschaft und Privatwirtschaft setzt („network developmental state“) und über Institutionen wie die Defense Advanced Research Projects Agency (DARPA) anwendungsorientierte Militärtechnologie oder über öffentliche „venture capital funds“ Technologie-Start-Ups finanziert werden (Wade 2012, S. 390 ff.). Der U.S. Innovation and Competition Act folgt diesem Ansatz, indem er etwa neben umfangreicher finanzieller Unterstützung für die Halbleiterindustrie auch ein Directorate for Technology, Innovation and Partnerships bei der National Science Foundation aufbaut, um die Forschung in High-Tech-Branchen zu fördern. Zusätzlich versucht die Biden-Administration, sich stärker mit ihren Verbündeten in Europa abzustimmen und so ein gemeinsames Vorgehen gegen China zu ermöglichen. Ein erster konkreter Schritt besteht hier in der Gründung eines Handels- und Technologierats, um sich in „wichtigen globalen Handels‑, Wirtschafts- und Technologiefragen zu koordinieren und die transatlantischen Handels- und Wirtschaftsbeziehungen auf der Grundlage gemeinsamer demokratischer Werte zu vertiefen“ (Europäische Kommission 2021a).

Während das zentrale Ziel der US-Politik einer Marktöffnung in China gescheitert ist, lassen sich infolge der Konflikte eine stärkere Entkoppelung (Decoupling) der US-Wirtschaft bzw. insbesondere des US-amerikanischen High-Tech-Sektors von der chinesischen Ökonomie und damit ein partieller Rückbau der Verflechtungen zwischen den beiden Wirtschaftsmächten beobachten.

## Die Rolle der EU: Gradueller Wandel und institutionelle Reform

Anders als in den USA kam es in der EU zu einer eher graduellen außenwirtschafts- und industriepolitischen Umorientierung. Der Ausgangspunkt war hier kein Regierungswechsel, sondern vielmehr ein Wandel des Chinabildes bei zentralen politischen Entscheidungsträger:innen und Unternehmensvertreter:innen. Ein wichtiger Akteur dieser Umorientierung war die deutsche Regierung, die später auch Akzente auf europäischer Ebene setzte, und insbesondere bei der französischen Regierung auf Unterstützung für einen neuen außenwirtschafts- und industriepolitischen Kurs gegenüber China traf. Ursächlich waren – neben dem aktiven Lobbying der US-Regierung für eine chinakritischere Haltung – drei Dynamiken: Erstens bewerteten europäische Wirtschaftsakteure wie der deutsche BDI das wirtschaftliche Umfeld in China neu. Bereits die genannte „Made in China 2025“-Strategie hatte durch die Förderung inländischer Unternehmen den Marktzugang nach China erschwert. Es entstand die Wahrnehmung, dass europäische Firmen auf neue Hindernisse bei ihrer Geschäftstätigkeit stoßen, während die Konkurrenz auf dem EU-Markt mit chinesischen Unternehmen wuchs (European Union Chamber of Commerce in China 2017). Zweitens führte der Zuwachs an chinesischen Direktinvestitionen in die EU zu Konflikten. Ein zentrales Ereignis war hier der „Kuka Moment“ im Jahr 2017, als der deutsche Industrieroboterhersteller Kuka durch den chinesischen Konzern Midea übernommen wurde. Die Übernahme verstärkte im deutschen Wirtschaftsministerium die Sorge vor dem Aufkauf von Hochtechnologie-Firmen durch chinesische Investoren. Drittens wurde die kritische Haltung durch die Diskussion über die Menschenrechtsverletzungen an der uigurischen Bevölkerung in Xinjiang und dem Sicherheitsgesetz in Hongkong verschärft. China beantwortete die EU-Sanktionen gegen chinesische Entscheidungsträger:innen Anfang 2021 mit Gegensanktionen von EU-Institutionen und Politiker:innen, was zur kritischeren Positionierung gegenüber China beitrug.

Die graduelle Reaktion ergab sich aus einer widersprüchlichen Interessenkonstellation: Viele europäische Unternehmen sind von den Geschäften auf dem chinesischen Binnenmarkt abhängig. Dies betrifft gerade die deutsche Automobilindustrie. Für den VW-Konzern war China beispielsweise der größte Einzelmarkt im Jahr 2020. Hinzu kommen die umfangreichen Direktinvestitionen, die europäische Unternehmen, anders als ihre US-amerikanischen Konkurrenten, auf dem chinesischen Markt getätigt haben. Gleichzeitig sehen sich verschiedene Industriesektoren, wie der deutsche Maschinenbau, verstärkt chinesischer Konkurrenz auf den globalen Märkten ausgesetzt (Matthes 2021; García-Herrero 2021). Die deutsche Regierung spielte deshalb auch eine Schlüsselrolle bei der Ausgestaltung der Regulierungsmaßnahmen auf EU-Ebene. Während in den USA die Widersprüche des Chimerica-Modells und neue chinesische Wettbewerber im sicherheitspolitisch sensiblen IT-Sektor den Ausgangspunkt einer heftigen politischen Reaktion mit parteiübergreifender Unterstützung bildeten, war der politische Handlungsspielraum in der EU begrenzt. Dies lag auch daran, dass die USA über größere Machtmittel verfügten, etwa ihren Technologievorsprung bei der Halbleiterproduktion. Die Ausgangslage in der EU war demnach defensiver, und es kam zu einer komplexen Diskussion um einen Maßnahmenkatalog („EU Toolbox“).

### Außenhandel

So setzte die EU bisher nur vereinzelt auf klassische handelspolitische Maßnahmen wie Anti-Dumpingzölle gegen China. Zwar wurde 2018 der Anwendungsbereich für (Straf‑)Zölle erweitert, aber Schutzzölle wurden nur für einzelne Produkte wie bestimmte Stahlerzeugnisse oder Graphitelektroden erlassen. Allerdings etablierte sich unter den politischen Entscheidungsträger:innen in der EU ähnlich wie in den USA ein Diskurs, bei dem die Kritik am chinesischen staatskapitalistischen Modell eine wichtige Rolle spielte. Während die EU eines der weltweit offensten Investitions- und Handelsregime habe, verweigere sich China einer solchen Öffnung und bevorzuge eigene Unternehmen nicht nur beim staatlichen Beschaffungswesen und im Wettbewerb auf dem Binnenmarkt, sondern unterstütze chinesische Unternehmen auch im Ausland. Dies führe zu unfairen Wettbewerbsvorteilen, so ein gängiger Vorwurf zentraler politischer und wirtschaftlicher Akteure in der EU (BusinessEurope 2020). Um das „Level-Playing-Field“^10^ wiederherzustellen, sind daher verschiedene handelspolitische Instrumente in Vorbereitung. Im Jahr 2021 stellte die EU-Kommission einen Verordnungsentwurf vor, um Drittstaatensubventionen mittels notifizierungsbasierter Untersuchungsinstrumente für Firmenzusammenschlüsse, Gründungen von Gemeinschaftsunternehmen und Angebote im öffentlichen Beschaffungswesen zu überprüfen. Dies gilt ab einer gemeinsamen Umsatzschwelle von 500 Mio. Euro bei Firmenzusammenschlüssen, 250 Mio. bei Staatsaufträgen und bei finanziellen Zuwendungen über 50 Mio. Euro durch Drittstaaten. Eine ähnliche Ausrichtung hat das International Procurement Instrument (IPI), dessen Umsetzung mit der Vorlage der neuen europäischen Industriestrategie 2020 vorangetrieben wurde. Das IPI zielt darauf, Wettbewerbsvorteile von staatlich geförderten Firmen aus Drittstaaten bei der Vergabe von Staatsaufträgen zu korrigieren. Falls keine Abkommen geschlossen wurden, besteht die Möglichkeit, Preisaufschläge oder Punktabzüge für Angebote über 5 Mio. Euro bei Waren und Dienstleistungen bzw. 15 Mio. Euro bei Bauleistungen und Konzessionen zu verhängen oder die Firmen komplett auszuschließen.

### Investitionen

Deutlicher war die Umorientierung im Bereich der Investitionsregulierung. So bemühte sich neben Frankreich und Italien insbesondere die deutsche Bundesregierung um eine EU-weite Regulierung. Zunächst verabschiedete sie jedoch verschiedene Novellen des Außenwirtschaftsrechts auf nationaler Ebene, die die sektorübergreifende Investitionskontrolle verschärften: Im Jahr 2017 etablierte die Bundesregierung eine Prüfschwelle für Beteiligungen ab 25 % durch Investoren aus Nicht-EU-Ländern und ein De-facto-Vetorecht, sofern die nationale Sicherheit oder öffentliche Ordnung gefährdet sind. Das Gesetz zielte auf Firmen im High-Tech-Bereich, in der Energieversorgung sowie der kritischen Infrastruktur. Bereits im Folgejahr wurde die Prüfschwelle auf Beteiligungen ab 10 % gesenkt und die Definition der Sektoren wurde – auch durch zusätzliche Änderungen im Investitionsprüfungsrecht 2020/2021 – erweitert. Die Bundesregierung nutzte nun bei kritischen Investitionen die Möglichkeit einer Selbstbeteiligung („Rückgriffoption“) durch die staatliche Förderbank KfW, wie sie etwa beim gescheiterten Übernahmeversuch des Übertragungsnetzwerkbetreibers 50Hertz durch den chinesischen Staatskonzern State Grid Corporation of China eingesetzt wurde.

Das Thema chinesische Investitionen wurde nun auch verstärkt auf EU-Ebene verhandelt. Hierbei war eine Trendumkehr erkennbar. Ursprünglich beabsichtigte die EU-Kommission mit dem seit 2014 verhandelten EU-China Comprehensive Investment Agreement (CAI) einen einheitlichen EU-Rechtsrahmen für Investitionsbeziehungen zu schaffen. Im Bereich des Investitionsregimes sollte das CAI Rechtssicherheit und besseren Marktzugang für europäische Investoren schaffen. Die Ratifizierung des Abkommens scheiterte jedoch zuletzt am Widerstand des EU-Parlaments. Um strategische Branchen besser schützen zu können, rückte somit die Investitionsregulierung in den Vordergrund. Bereits im Jahr 2019 wurde auf EU-Ebene eine Verordnung zur Schaffung eines Rahmens für das Screening von ausländischen Direktinvestitionen verabschiedet. Geplante Investitionen in Mitgliedstaaten mit Screening-Mechanismus müssen nun auf europäischer Ebene gemeldet werden und durchlaufen ein Genehmigungsverfahren (Europäische Kommission 2020; Zwartkruis und De Jong 2020). Die Motivation für diese Maßnahmen waren – wie auch in Deutschland – zahlreiche Übernahmen europäischer Firmen durch chinesische Unternehmen im Hochtechnologiebereich. Das Screening-Verfahren ist jedoch ein weiches Instrument. Bisher konnten sich Deutschland, Frankreich und Italien innerhalb der EU mit der Idee einer europaweiten Regulierungsbehörde im Stil des US-amerikanischen CFIUS nicht durchsetzen (Chan und Meunier 2021).

### Hochtechnologie-Konkurrenz

Anders als die USA verhängte die EU keine Sanktionen gegen chinesische High-Tech-Unternehmen und konnte auch beim Thema der Nutzung der 5G-Netzwerkinfrastruktur von Huawei keinen einheitlichen Kurs entwickeln. Dennoch kam es auf nationaler Ebene hier zu deutlichen Eingriffen. Verschiedene Mitgliedstaaten wie Frankreich oder Polen schlossen Huawei-Komponenten de facto komplett aus den einheimischen 5G-Mobilfunktnetzen aus, während andere Länder wie Italien oder Deutschland Teilausschlüsse in einzelnen Bereichen des Netzes umsetzten. Nur wenige Länder wie Finnland oder Tschechien implementierten keine Maßnahmen (Poggetti 2021). Konflikte um Hochtechnologie traten auch im Rahmen der Bekämpfung der COVID-19-Pandemie zutage, insbesondere bei der Frage nach der Freigabe von Patenten und der gegenseitigen Zulassung von Impfstoffen. So scheiterte der von Indien und Südafrika bei der WTO eingebrachte Antrag zur zeitweisen Aussetzung der geistigen Eigentumsrechte auf Impfstoffe und weitere Präparate gegen COVID-19 lange Zeit vor allem am Widerstand Deutschlands und der EU. Dies wurde damit begründet, dass ein Transfer der modernen mRNA-Technologie für Impfstoffe nach China verhindert werden soll (Köncke und Schmalz 2022).

Die High-Tech-Konkurrenz wurde insbesondere durch neue industriepolitische Initiativen der deutschen Bundesregierung angeheizt. Im Februar 2019 stellte Wirtschaftsminister Altmaier die „Nationale Industriestrategie 2030“ (später: „Industriestrategie 2030“) vor, in der China als zentraler Konkurrent benannt wurde und Staatsinterventionen gefordert wurden (Schneider 2020). Dem war ein Grundsatzpapier zu China durch den Bundesverband der Deutschen Industrie (BDI 2019) vorausgegangen, dessen wesentliche Aussage war, dass das „Modell Deutschland“ im „Systemwettbewerb“ mit China einer Reform bedürfe. Das Papier traf auf große Resonanz, zunächst in der Politik und später in der Wirtschaft. Als Folge rückten verschiedene Verbände, Parteien und Wirtschaftsakteure, die das neoliberal ausgerichtete Projekt der EU-Integration seit den 1990er-Jahren maßgeblich getragen hatten, von einigen zentralen Positionen ab. Sie unterstützten fortan eine stärker neo-merkantilistische Ausrichtung und Staatsinterventionen in der Industriepolitik, um die Wettbewerbsposition deutscher und europäischer Konzerne gegenüber China (und den USA) zu verbessern (Schneider 2020). So kam es in der EU zu einer graduellen industriepolitischen Reorientierung. Begünstigt wurde dieser Wandel durch die unter Druck stehende EU-Wirtschaft, die sich ohnehin aufgrund des doppelten Übergangs aus digitaler und ökologischer Transformation gegen die chinesische Konkurrenz neu ausrichten musste (Landesmann und Stöllinger 2020).

Dabei erwiesen sich gängige Instrumente, insbesondere der Wettbewerbspolitik und der Fusionskontrolle als dysfunktional. Dies wurde an der gescheiterten Fusion der Bahnsparten von Alstom und Siemens offensichtlich. Der Fusionsversuch, der auf die Schaffung eines „Eurochampions“ zielte, um gegenüber dem chinesischen Eisenbahnhersteller CRRC konkurrenzfähig zu bleiben, scheiterte am Veto der EU-Wettbewerbskommissarin Margarethe Vestager. Hieraus entstand eine lebhafte Reformdiskussion der EU-Fusionskontrollverordnung. Die EU müsse Marktdominanz auch vor dem Hintergrund der Weltmarktposition definieren und nicht länger nur hinsichtlich der innereuropäischen Position (Pang 2020). Die Forderungen nach einer ambitionierteren und langfristig ausgerichteten EU-Industriepolitik wurden dabei im Wesentlichen von der deutsch-französischen Achse vorangetrieben (ebd.; BMWi 2019). Im Mai 2021 legte die Europäische Kommission eine Aktualisierung der europäischen Industriestrategie vor, die Lehren aus der COVID-19-Pandemie inkorporierte. Zentrales Ziel ist nun die Reduktion strategischer Abhängigkeiten von importierten Produkten – u. a. aus China – in neu definierten „sensiblen Ökosystemen“ mittels Diversifizierung von Lieferketten (Europäische Kommission 2021b). Dabei spielen Industrieallianzen eine wichtige Rolle, insbesondere in Bereichen der „grünen“ und „digitalen“ Transformation, in denen Innovationen durch den Markt ausbleiben (Belitz et al. 2021, S. 10). So bestehen heute bereits EU-Industrieallianzen für wirtschaftlich wichtige Rohstoffe mit Beschaffungsrisiko, Wasserstoff, Batteriezellfertigung, Prozessoren und Halbleiter sowie Industriedaten, Edge und Cloud. Diese Industrieallianzen werden durch die grenzüberschreitenden Industriekonsortien „Important Projects of Common European Interest“ (IPCEI) als zentraler Umsetzungsmechanismus der EU-Industriestrategie ergänzt und teilweise weitergeführt. Hierfür werden nationale öffentliche Finanzmittel der Mitgliedstaaten mit Eigenbeteiligungen der teilnehmenden Firmen gebündelt und durch die Europäische Kommission im Sinne beihilferechtlicher Regelungen genehmigt. Die IPCEIs sind dabei so ausgestaltet, dass sie den von der europäischen Wettbewerbspolitik maximal zulässigen Spielraum für ambitionierte industriepolitische Maßnahmen ausschöpft (ebd.). Denn das europäische Beihilferecht schränkt den Spielraum für eine vertikale Industriepolitik und aktiv-gestalterische Staatsinterventionen und Subventionen ein (Schneider 2020). Der Aufstieg chinesischer Konzerne übt also auch Reformdruck in diesem Politikfeld aus.

Die außenwirtschaftlichen und industriepolitischen Reaktionen in der EU liefen somit nach einem anderen Muster ab als in den USA. Aufgrund einer widersprüchlichen Interessenkonstellation und einer restriktiven Wettbewerbsordnung in der EU kam es zu einer defensiveren Reaktion, gleichzeitig trug der Aufstieg chinesischer Unternehmen aber zu institutionellen Reformen in der EU bei. Zudem hat die EU-Kommission vor dem Hintergrund der Abhängigkeit im Bereich von Technologie (Digitalwirtschaft) und Grundgütern (medizinische Ausrüstung) nunmehr das Ziel einer „offenen strategischen Autonomie“ formuliert – sowohl mit Blick auf China als auch die USA. Auch wenn verschiedene Reforminitiativen neue Barrieren in der Außenwirtschaft errichten, sind sie bisher jedoch keine Triebkräfte eines umfangreichen Rückbaus der Wirtschaftsbeziehungen mit China.

## Umkämpfte Globalisierung: Neue Konfliktlinien in der Weltwirtschaft

Der Aufstieg des hybriden Parteistaatskapitalismus in China und die Internationalisierung chinesischer Unternehmen haben zu neuen Konfliktlinien in der Weltwirtschaft geführt. Der Modus der Globalisierung ist zunehmend umkämpft. Sowohl die USA als auch die EU haben außenwirtschafts- und industriepolitische Maßnahmen ergriffen, um die Stellung ihrer Hochtechnologie-Unternehmen im internationalen Wettbewerb gegen die chinesische Konkurrenz zu stützen und die Akquisition von kritischer Infrastruktur und Technologie durch chinesische Unternehmen zu verhindern. Dabei griffen sie zu unterschiedlichen Mitteln. Während die USA auf eine aggressive Handels- und Sanktionspolitik und Technologieförderung setzten, trieb die EU graduelle Reformen des Investitions- und Handelsregimes und der Industriepolitik voran. Diese Herangehensweisen ergeben sich aus unterschiedlichen Kräfteverhältnissen und Weltmarkteinbindungen. Während in den USA ein parteiübergreifender Konsens für eine harte Gangart und für weitreichende wirtschaftspolitische Sanktionsmöglichkeiten gegenüber China besteht, ist die Interessenkonstellation in der EU sehr viel widersprüchlicher. Zudem sind zentrale europäische Industriesektoren wie der Maschinenbau, die Automobil- und die Chemieindustrie vom chinesischen Markt abhängig.

Die unterschiedlichen Maßnahmen haben dazu beigetragen, dass sich die wirtschaftlichen Verflechtungen zwischen China und den USA sowie zwischen China und der EU abweichend entwickelt haben. In den von uns untersuchten Bereichen lässt sich ein Rückbau oder zumindest eine Stagnation der US-amerikanisch-chinesischen Handels- und Investitionsbeziehungen beobachten: Trotz einer deutlichen Erholung nach dem Pandemiejahr 2020 bewegte sich der bilaterale Außenhandel zwischen den USA und China im Jahr 2021 mit 657,4 Mrd. US-Dollar immer noch leicht unter dem Wert 658,7 Mrd. US-Dollar von 2018 (United States Census Bureau 2022). Noch deutlicher ist diese Dynamik bei den ausländischen Direktinvestitionen (ADI). Hier ist ein langsamer Rückgang der US-Direktinvestitionen in China seit 2016 zu beobachten, und ein deutlicher Fall der chinesischen ADI in den USA seit 2017 (Rhodium Group 2022a). Im Hochtechnologie-Sektor äußern sich die US-Sanktionen in veränderten Marktanteilen. ZTE verlor etwa seinen 11 %-Anteil am US-Smartphone-Markt, während der zeitweilige Weltmarktführer Huawei heute nicht mehr unter den globalen Top 10 zu finden ist.

In der EU führte die graduelle Neuausrichtung des Handels- und Investitionsregimes bislang zu keinem Bruch in den Wirtschaftsbeziehungen mit China. Der Außenhandel zwischen den beiden Wirtschaftsblöcken stieg in den vergangenen Jahren sogar deutlich an: von 530,5 Mrd. Euro im Jahr 2018 auf 695,5 Mrd. Euro im Jahr 2021 (Eurostat 2022). Im Hochtechnologie-Bereich wie beim Smartphone-Verkauf konnten andere chinesische Hersteller wie Xiaomi ihre Marktanteile auf Kosten von Huawei ausbauen. Lediglich im Bereich der ADI zeigt sich ein ähnliches Bild wie in den USA. Auch in der EU ist seit 2016 ein kontinuierlicher Rückgang chinesischer Direktinvestitionen zu beobachten (Rhodium Group 2022b). Allerdings wurden in der EU verschiedene wirtschaftspolitische Initiativen wie das IPI, die sich gegen China richten, noch nicht umgesetzt. Folglich sind zukünftig weitere Barrieren für chinesische Konzerne zu erwarten.

Für China bedeuteten diese neuen Konflikte, dass die staatsgeleitete Globalisierungsstrategie in den traditionellen Industrieländern an Grenzen gerät. Zu groß sind hier die Widerstände der politischen Entscheidungsträger:innen in den USA und der EU aufgrund von (sicherheits-)politischen Bedenken und zunehmender wirtschaftlicher Konkurrenz. China scheint darum die Ausrichtung auf Drittmärkte in Asien und anderen Regionen des globalen Südens zu forcieren. Diese Orientierung, die sich im Grunde schon nach der Finanz- und Währungskrise 2008/09 mit der Erschließung neuer Exportmärkte und den großen Investitionsvorhaben im Rahmen der BRI andeutete, hat nun durch neue Projekte, wie etwa das seit 2022 in Kraft getretene Handelsabkommen Regional Comprehensive Economic Partnership in der Asienpazifikregion (ASEAN-Länder, China, Japan, Korea, Australien und Neuseeland), weiter Fahrt aufgenommen. Gleichzeitig setzt die Regierung mit dem eingangs erwähnten 14. Fünfjahresplan auf einen Ausbau des Binnenmarkts und technologisches Upgrading. Diese Orientierung bedeutet keinen Rückzug vom Weltmarkt, sondern zielt vielmehr darauf, Chinas Rolle in der Weltwirtschaft zu verändern. Neben der industriellen Aufwertung werden Wertschöpfungssysteme in der Binnenwirtschaft durch die Förderung von lokalen Zulieferstrukturen ausgebaut. Dies schließt nicht aus, dass auch weiterhin Investitionen von ausländischen Unternehmen willkommen sind. So lockerten die chinesischen Behörden etwa den Joint-Venture-Zwang in der Automobilindustrie für Hersteller wie Tesla, um die Ansiedlung von innovativer Wertschöpfung zu fördern, oder ermöglichten neue Partnerschaften zwischen US-Investment- und chinesischen Staatsbanken auf den heimischen Finanzmärkten. Die Konflikte mit den USA und der EU haben demnach zwar eine Neuausrichtung der chinesischen staatsgeleiteten Globalisierung in Richtung des Aufbaus resilienter Wertschöpfungssysteme und die Erschließung neuer Märkte katalysiert, diese bisher aber nicht ausgebremst.

Die staatsgeleitete Globalisierungsstrategie bleibt jedoch umkämpft. Ein großes Risiko für China besteht darin, dass sich die Konflikte mit den westlichen Ländern ausweiten und sich die Barrieren nicht nur zwischen China, der EU und den USA vertiefen, sondern sich neue wirtschaftliche Blöcke herausbilden. Sehr deutlich wird dies nicht nur in den Debatten um ein „reshoring“ von Wertschöpfungsketten in den USA und der EU, die durch den Ausbruch der COVID-19-Pandemie einen Schub bekamen (Raza et al. 2021), sondern auch am Fall der russischen Invasion in der Ukraine. Die massiven Sanktionen, die die USA, die EU und ihre Verbündeten gegen Russland verhängt haben, gingen mit dem Ende der Geschäftstätigkeit vieler transnationaler Konzerne in dem Land einher. Dies zieht beinahe zwangsläufig eine stärkere Anbindung Russlands an China nach sich, da chinesische Unternehmen in Branchen wie der Automobilindustrie bereitwillig die Marktanteile übernehmen. Allerdings zeigt die jüngste Warnung der US-Regierung Biden an chinesische Konzernen, die ähnlich wie Huawei im Fall Iran die Sanktionen gegen Russland umgehen und auf einer schwarzen Liste für US-Export- und Investitionsbeschränkungen enden könnten, dass sich Chinas Partei- und Staatsführung auf einem schmalen Grat bewegt. Denn es drohen zudem neue Konflikte, die eine Abkopplung Chinas von den USA und auch der EU vorantreiben könnten. Eine stärkere Fragmentierung der Weltwirtschaft, bei der regionale Blöcke – z. B. ein westlich-japanisches Konglomerat versus einen chinesisch-russischen Block – mit jeweils eng verflochtenen Wirtschaftskreisläufen und technologischen Innovationszentren entstehen könnten, ist zumindest ein reales Entwicklungsszenario. Allerdings besteht hier bisher kein Risiko einer umfangreichen Deglobalisierung, wie sie in früheren Umbruchsphasen im kapitalistischen Weltsystem zu beobachten war (Arrighi und Silver 1999). Vielmehr könnte eine Zuspitzung des Systemkonflikts zwischen hybridem Parteistaatskapitalismus in China und westlichen liberalen Kapitalismen zu einer „chaotischen Melange“ (McNally 2020) von institutionellen Regulierungen und Einflusssphären zwischen den USA und ihren traditionellen Verbündeten, wie der EU, sowie zwischen China und seinen Partnern im eurasischen Raum führen. Ob die umkämpfte Globalisierung zu einer weitgehenden Restrukturierung der Weltwirtschaft beitragen wird, bleibt daher abzuwarten.

## Footnotes

1 Der Artikel ist im Projekt JRT02 „Kampf oder Konvergenz der Kapitalismen. Eigentumskonflikte um chinesische Direktinvestitionen in Deutschland und der Europäischen Union“ im Rahmen des von der DFG finanzierten Sonderforschungsbereichs 294 „Strukturwandel des Eigentums“ (Universität Erfurt/Friedrich-Schiller Universität Jena) entstanden. Während die Daten für den Abschnitt zu den USA in Abschnitt 4 aus Dokumenten, Sekundärliteratur und offiziellen Statistiken entnommen wurden, haben wir für den Teil zur EU in Abschnitt 5 zusätzlich auf Material aus der empirischen Forschung im Rahmen des Projekts JRT02 und damit auf insgesamt 21 leitfadengestützte Expert:inneninterviews mit Verbändevertreter:innen, Abgeordneten, Regierungsvertreter:innen und Think Tanks zurückgegriffen, die zum Thema der außenwirtschafts- und industriepolitischen Reaktion der EU und Deutschlands auf den Aufstieg Chinas geführt wurden. Die Interviewdaten wurden mit qualitativer Inhaltsanalyse ausgewertet. Die Interviewdaten ermöglichen es, einige noch in der Ausgestaltung befindliche politischen Initiativen besser bewerten und kontextualisieren zu können.

2 Das Argument zur endemischen Korruption kann sicherlich auch kritisch gesehen werden, da der Parteistaat in der Xi Jinping-Periode gezielt gegen Korruption unter Partei- und Staatsfunktionären vorgeht.

3 Exemplarisch ist hier die Zwangsübernahme des privaten Versicherungskonzerns Anbang durch den chinesischen Staat im Februar 2018. Drei Monate später wurde der Unternehmensgründer Wu Xiaohui wegen Betrug und Veruntreuung zu einer Haftstrafe von 18 Jahren verurteilt, im September 2020 löste die Regulierungsbehörde den Konzern auf. Ein ähnliches Vorgehen gab es gegen Xiao Jianhua, der 2017 verschwand und mutmaßlich festgehalten wird. Seine Unternehmensgruppe, die Tomorrow Group, wurde von Regierungsinstitutionen übernommen und restrukturiert.

4 So hat beispielsweise das Onlinehandelsunternehmen Alibaba kürzlich bekanntgegeben, bis 2025 insgesamt 15,5 Mrd. US-Dollar in Kampagnen zur „Erhöhung des allgemeinen Wohlstands“ zu investieren. Hierzu zählen insbesondere die Subventionierung kleiner und mittelständischer Unternehmen und die Verbesserung des Versicherungsschutzes von „Gig Economy“-Arbeitskräften. Wenige Monate zuvor wurde der Konzern wegen Missbrauchs seiner dominanten Marktposition mit einer Strafe von 2,75 Mrd. US-Dollar sanktioniert.

5 Anders als bei staatseigenen Unternehmen verfügen Parteigruppen in privaten Unternehmen jedoch über keine formalen Entscheidungsbefugnisse. Vielmehr übernehmen sie beratende Funktionen und sind dafür zuständig, Geschäftsrisiken zu erkennen und finanzielle Stabilität zu gewährleisten.

6 In diesem Artikel beziehen wir uns auf die Globalisierungsdefinition des britischen Geografen David Harvey (1990, S. 240 ff.) demzufolge Globalisierung als eine „Zeit-Raum-Kompression“ verstanden werden kann. Technologischer Fortschritt und wirtschaftliche Liberalisierung überwinden räumliche Distanzen und führen zu neuen grenzüberschreitenden Interaktionen, wie im Fall von ökonomischer Globalisierung bei Handel, Investitionen oder Finanzflüssen. Wirtschaftliche De-Globalisierung impliziert hingegen einen Rückgang solcher Verflechtungen.

7 Auch die Gründung der Asian Infrastructure Investment Bank, die 2015 auf Initiative Chinas errichtet wurde, ihren Hauptsitz in Peking hat und regionale Infrastrukturprojekte in Asien finanziert, ist in diesem Zusammenhang zu nennen.

8 Hierbei war bezeichnend, dass es parteiübergreifende Initiativen gab, u. a. unterstützt von dem republikanischen Senator Marco Rubio und der Demokratin Elizabeth Warren, die sogar über die Maßnahmen hinausgingen, die die Regierung Trump anstrebte. Diese Zusammenarbeit blieb auch in der Amtsperiode Biden erhalten.

9 Siehe die Daten des US Department of Commerce, Bureau of Industry and Security: https://www.bis.doc.gov/index.php/documents/regulations-docs/2326-supplement-no-4-to-part-744-entity-list-4/file.

10 Der Begriff „Level-Playing-Field“ stammt aus der Handelspolitik und bezieht sich auf gleiche Standards und Regeln, sodass Firmen in einem Land keinen Wettbewerbsvorteil gegenüber Firmen aus anderen Ländern erhalten.
